# Carotid artery stenosis: to infinity and beyond

**DOI:** 10.1590/0004-282X-ANP-2022-E004

**Published:** 2022-05-18

**Affiliations:** 1 Universidade Federal do Paraná, Curitiba PR, Brazil.; 2 Academia Brasileira de Neurologia, São Paulo SP, Brazil.; 3 Executive Member of the Neurosonology Committee, Neurosonology Speaciality Research Group, World Federation of Neurology, London, UK.; 4 Miami University, Miller School of Medicine, Department of Neurology Clinical Translational Research Division, Miami, EUA.; 5 University of Arizona, Evelyn F. McKnight Brain Institute, Arizona, EUA.

**T**hrombosis or embolism originating from large artery atherosclerosis causes 15% of the ischemic strokes with a transient or permanent functional deficit. Primary stroke prevention is based on the control of lifestyle-related risk factors and optimal medical therapy. Patients with a significant atherosclerotic narrowing in their carotid artery can also benefit from an additional surgical or endovascular intervention to reduce the risk, having in mind that atherosclerosis is a systemic disease. We all know that the degree of stenosis is a relevant risk factor of ipsilateral ischemic stroke, and this criterion has been used to select patients in randomized clinical trials. Several clinical trials have been performed to find a cut-off for stenosis and classify the risk of symptomatic and asymptomatic carotid artery disease. However, after the introduction of the so-called ‘aggressive medical treatment’, there is a lack of long-term results regarding this new medical approach. On the other hand, some ischemic strokes associated with carotid artery disease result from hypoperfusion, in addition to a vulnerable atherosclerotic plaque embolization in which no clinical treatment can solve the issue. We also know that these two mechanisms may act synergistically, and a more profound investigation can make the difference in treating this setting. Routine and more advanced imaging modalities may provide information on the underlying mechanism involved in the ipsilateral ischemic stroke. New concepts about intracranial hemodynamic status and the morphology of the plaque have been discussed, in addition to many neuroimaging modalities to better understand the pathophysiology involved in each situation. Management is an ongoing debate and, most of the time, treatment to prevent an ischemic or recurrent stroke involves some individualized decisions^1,2^.

Cerebral ischemia will arise due to reduced oxygen being delivered to the tissue and can occur because of reduced oxygen delivery with or without arterial steno-occlusion. Compromised cerebral perfusion pressure (CPP) occurs in phases, where compensatory mechanisms can be evaluated. When reduced oxygen delivery starts, vasodilation of pial arterioles will ensue, resulting in decreased vascular resistance and increased inflow. In some cases, these tissue-level compensations may be sufficient to ensure the necessary supply of oxygen, but chronic and increased reductions may cause these vessels to approach their dilation limit or exhaust the cerebrovascular reserve capacity. The term cerebrovascular reactivity (CVR) reflects the ability of the blood vessels to dilate to match tissue blood supply to the increased demand and can be investigated by measuring the change in cerebral blood flow (CBF) or cerebral blood volume (CBV) induced by vasodilation. This is obviously not new, and several imaging methods can assess hemodynamic changes to better understand in which stage brain perfusion is facing extracranial stenosis^3,4^. In 1998, Grub and colleagues already demonstrated in the St. Louis Carotid Occlusion study that elevated OEF (oxygen extract fraction) is an independent predictor of stroke in patients with carotid occlusion with an ipsilateral ischemic stroke rate at two years of 5.3% in 42 patients with normal OEF and 26.5% in 39 patients with increased OEF (p=0.004)^5^. Since then, this result has been reproduced in many studies, with several methodologies and neuroimaging modalities. Nevertheless, although these data are recognized as important, this approach could not be established in guidelines mainly because of the unavailability of methods and because of the high cost to screen all patients with carotid stenosis. More recently, neurovascular coupling has been studied following the same principle of hemodynamic response after metabolic demand. The relationship between local activity and subsequent changes in cerebral blood flow can be considered impaired or not^6^.

The article published in the April 2022 issue of *Arquivos de Neuropsiquiatria* shows the response to a motor task protocol as a provocative vasodilatory/vasoreactivity TCD test comparing patients with unilateral symptomatic carotid stenosis (three groups according to the stenosis degree) and health subjects^7^. The study was analyzed considering the degree of stenosis and the asymptomatic and symptomatic sides. They showed an association between impaired CVR and carotid occlusion when compared with the healthy population, and some additional findings concerning the pulsatility index and the sides of stenosis. Although the study design included a small number of patients and did not perform micro embolus monitoring, the authors offer us a simple and accessible tool to access hemodynamic information that can improve our decision-making. Despite the completion of several multi-center trials, the management of carotid stenosis remains in flux and any additional information can help make more reliable individual decisions. Dr. Aysel Milanlioglu et al.^7^ demonstrated that TCD can provide information about cerebral hemodynamic status in real-time through an easy and affordable process. TCD with stimulus represented by motor tasks can inform a ‘valid number’ to explore cerebral autoregulation through the compensatory mechanisms, including vasoreactivity and functional reserve. They argue that it is possible to more effectively determine who the high-risk patients are. The authors found a good correlation between a low reserve and a high degree of stenosis reinforcing this concept. Considering that stenosis is dynamic and includes other variables, individual hemodynamic status data should be guaranteed when monitoring in real-time and should be repeated several times during follow-up. An association with neuroimage markers, like watershed or border zone, can also provide a full understanding of its relationship in symptomatic patients.

Future validation of these method findings could lead to powerful biomarkers of cerebrovascular health and create a different way of thinking in a setting of cerebrovascular disease.

## References

[1] Del Brutto VJL, Gornik LH, Tatjana Rundek T (2020). Why are we still debating criteria for carotid artery stenosis?. Ann Transl Med.

[2] Abbott AL, Brunner AM, Giannoukas A, Harbaugh RE, Kleinig T, Lattanzi S (2020). Misconceptions regarding the adequacy of best medical intervention alone for asymptomatic carotid stenosis. J Vasc Surg.

[3] Purkayastha S, Farzaneh S (2012). Transcranial doppler ultrasound: technique and application. Semin Neurol.

[4] McDonnell MN, Berry NM, Cutting MA, Keage HA, Buckley JD, Howe PRC (2013). Transcranial Doppler ultrasound to assess cerebrovascular reactivity: reliability, reproducibility, and effect of posture. PeerJ.

[5] Grubb RL, Derdeyn CP, Fritsch SM, Carpenter DA, Yundt KD, Videen TO (1998). Importance of hemodynamic factors in the prognosis of symptomatic carotid occlusion. JAMA.

[6] Juttukondaa MR, Donahuea MJ (2019). Neuroimaging of vascular reserve in patients with cerebrovascular diseases. Neuroimage.

[7] Milanlioglu A, Yaman A, Kolukisa M, Asil T (2022). Evaluation of cerebral hemodynamic status in patients with unilateral symptomatic carotid artery stenosis during motor tasks, through use of transcranial Doppler sonography. Arq Neuropsiquiatr.

